# Comparison of long-term clinical and radiologic outcomes of AperFix and fixed loop device fixation in anterior cruciate ligament reconstruction: A retrospective study

**DOI:** 10.1097/MD.0000000000041199

**Published:** 2025-01-17

**Authors:** Sehmuz Kaya, Necip Guven, Yunus Can Unal, Sezai Ozkan, Cihan Adanas, Tulin Turkozu, Ferhat Danisman, Ulan Ismailov, Abdulrahim Dundar, Zulkuf Akdemir, Mehmet Ata Gokalp

**Affiliations:** a Department of Orthopaedics and Traumatology, Faculty of Medicine, Van Yuzuncu Yil University, Van, Turkey; b Department of Orthopaedics and Traumatology, Van Education And Research Hospital, Van, Turkey; c Department of Orthopaedics and Traumatology, Sanliurfa Education And Research Hospital, Sanliurfa, Turkey; d Department of Orthopaedics and Traumatology, Faculty of Medicine, Hitit University, Corum, Turkey; e Department of Radiology, Faculty of Medicine, Van Yuzuncu Yil University, Van, Turkey.

**Keywords:** anterior cruciate ligament, anterior cruciate ligament reconstruction, equipment design, knee injury

## Abstract

Anterior cruciate ligament reconstruction aims to improve knee stability and range of motion. The AperFix system consists of polymer components, and fixed-loop fixation is an established endoscopic technique. Our aim in this study was to compare the long-term clinical and radiological results of AperFix and fixed-loop fixation and to prove that the long-term results of the AperFix fixation method are at least as good as those of the fixed loop device. This retrospective study included 109 patients who underwent primary anterior cruciate ligament reconstruction using single bundled hamstring tendon grafts. Patients under 16 years of age, patients with incomplete follow-up, bilateral or other ligament injuries, inflammatory arthropathy, previous knee surgery, or concurrent meniscal treatment were excluded. Participants were divided into 2 groups according to femoral fixation methods: AperFix fixation (group 1, n = 55) and fixed loop device fixation (group 2, n = 54). All operations were performed by senior surgeons under general or spinal anesthesia. Postoperative rehabilitation started on day 1 and allowed patients to resume normal activities at 6 months. Outcomes were evaluated during follow-up, including knee range of motion, clinical scores [Lysholm, Cincinnati, Tegner, and International Knee Documentation Committee] and radiographic measurements of femoral tunnel width and length. Measurements were performed by 2 orthopedic surgeons to ensure reliability. This study evaluated 109 patients (55 in group 1, 54 in group 2) and found no statistically significant differences in demographic variables such as age, sex, body mass index, follow-up duration, or side distribution. Clinical outcomes, including anterior drawer test, Lachman test results, knee flexion-extension degrees, and Lysholm, Cincinnati, Tegner, and International Knee Documentation Committee scores, were similar between the groups (*P* > .05). Complications occurred in 8 cases (rerupture, infection, and deep vein thrombosis), with no significant correlation to the fixation method used (*P* = .506). Radiographic analysis revealed no significant differences in femoral tunnel width or length between the groups (*P* > .05). In our current study, no meaningful disparity was found between the AperFix and fixed loop device methods in terms of long-term clinical outcomes. As there are no long-term studies on the results of AperFix fixation in the literature, more studies on this subject are needed.

## 1. Introduction

The anterior cruciate ligament (ACL) primarily serves as a stabilizer in the knee joint, limiting the forward movement, and internal rotation of the tibia. ACL injuries result in anterior and rotational instability^[1]^ and are usually caused by noncontact trauma to the knee joint in the semiflexion and valgus position.^[2–4]^ The gold standard in the treatment of ACL tear is ACL reconstruction.^[5,6]^ In the literature, consensus has yet to be reached on the best fixation system for ACL reconstruction using tendon grafts.^[7,8]^

**Figure 1.**
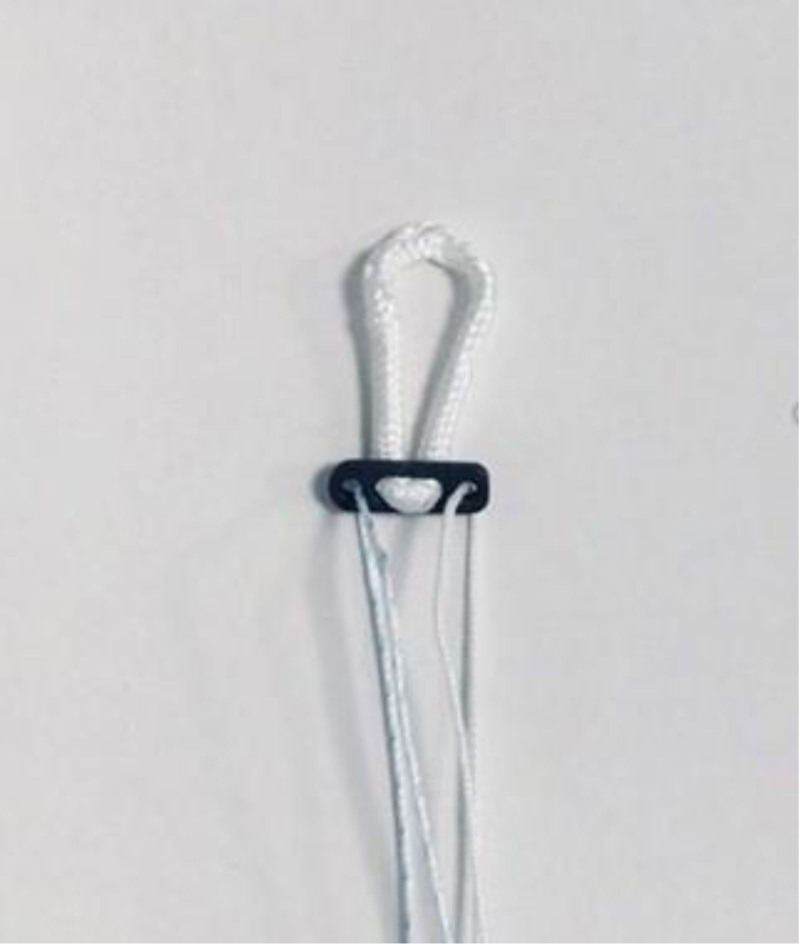
Fixed loop device femoral fixation system (Artrotek Med., Adana, Turkey).

**Figure 2.**
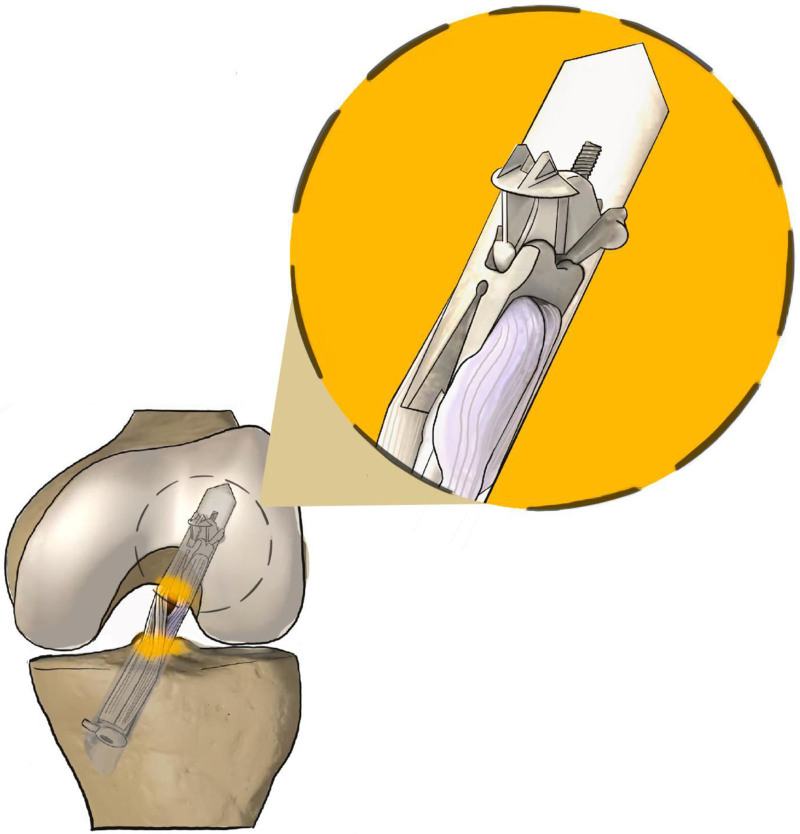
Cayenne AperFix (Cayenne Medical, Inc, Scottsdale, Arizona). Software: Procreate, Version: 5.3.7, Manufacturer: Savage Interactive Pty Ltd.

One of these methods, the fixed loop device, consists of an extra-articular device consisting of a metallic button and a polyurethane strip. The button is supported by the external cortical bone while the ribbon links the graft and the button together. The fixed loop device maintain the graft in tension to maintain the bone-graft interface for healing^[9,10]^ (Fig. 1). The AperFix system, which is less common in the literature, has been an option for ACL reconstruction since 2007.^[11]^ The AperFix femoral component comprises 2 arms, an upper and a lower one, and features a central hole. It accommodates 2 tendon bundles through the distal central hole. Upon insertion of AperFix into the femoral tunnel, the proximal arms extend outwards and attach directly to the spongy bone, while the distal arms project both forward and backward. This configuration allows for circular compression of the tendon bundles against the tunnel walls^[11–16]^ (Fig. 2).

Since there is no consensus in the literature about which femoral fixation method should be preferred and there are no data on the long-term results of the AperFix method, we aimed to contribute to the literature on this subject.

## 2. Materials and methods

This study was approved by the Ethics Committee (consent no: 2022-21-02). Given the retrospective nature of the study and anonymized data analysis, we did not seek informed consent from the participants. This study was not funded by any external source. We retrospectively evaluated 453 patients who underwent primary ACL reconstruction using a single bundle tendon graft. Patients who did not attend the follow-up examination, whose records could not be accessed, whose X-rays were not taken properly, who underwent knee surgery for a different reason, who were under 16 years of age, who had inflammatory arthropathy, and who had bilateral knee injuries, other knee ligament injuries (such as anterolateral ligament injury, posterior cruciate ligament, medial collateral ligament, or lateral collateral ligament) or a history of previous knee surgery were excluded. Concurrent meniscal treatment (meniscectomy or meniscal repair) was considered as exclusion criteria. All patients who attended regular follow-up examinations, underwent unilateral primary ACL reconstruction and were older than 16 years were included in the study. We included 109 patients who met the criteria for the study, and they were divided into 2 groups based on the femoral fixation method applied during their operations. AperFix fixation was performed in 55 patients (group 1) and fixed loop device fixation in 54 patients (group 2). Patients admitted to the outpatient clinic with knee trauma were diagnosed with ACL rupture according to their physical examination and subsequent MR images, and were scheduled undergo surgery.

### 2.1. Surgical technique

All surgical procedures were performed under general or spinal anesthesia by senior surgeons. In both patient groups, the operation was initiated using a tourniquet, and single bundle reconstruction was performed with the hamstring tendon. In both the methods, a vertical skin incision of approximately 3 cm was made medial to the tibial tuberosity. An inverted L-shaped incision is made over the pes anserinus tendon. The gracilis and semitendinosus tendons were removed and the sartorius fascia was repaired.

In the fixed loop method, first, the femoral tunnel was opened anatomically through the anteromedial portal at the 11 and 1 o’clock positions on the right and left sides. Then, the tibial guide was placed 2 cm medial to the tibial tuberosity at an angle of 55° to the long axis of the tibia, with the exit point 6 to 7 mm anterior to the posterior cruciate ligament. The tibial tunnel was drilled using a suitable drill. A single bundle of hamstring grafts was prepared. It was placed in the fixed loop device and pulled towards the femoral tunnel, and the device was placed in the femoral cortex. The tendon was fixed to the tibial tunnel by using a tibial screw. The vast majority of the tibial implants used were 9 mm. A u-nail was also used to increase stability.

In the AperFix method, the tibial tunnel is opened first. The guide was inserted 2 cm medial to the tibial tuberosity at an angle of 55° to the long axis of the tibia, with an exit point of 6 to 7 mm in front of the posterior cruciate ligament. The tibial tunnel was drilled using a drill suitable for the graft size. The femoral guide was then inserted through the tibial tunnel using a femoral offset guide selected according to the graft thickness, with the knee in hyperextension. Nine millimeter AperFix femoral implant was used for the femoral fixation. The single-loop hamstring autograft was placed in the femoral tunnel and inserted into the femoral tunnel through the tibial hole with an auxiliary inserter arm. After the safety pin on the lever was removed, the graft was fixed to the tunnel using an implant insertion button. During tibial fixation, AperFix polyetheretherketone polymer tibial implant sheaths were inserted into the tibial tunnel. The vast majority of the tibial implants used were 9 mm. The AperFix polyetheretherketone polymer tibial screw was inserted into the tibial sheaths at controlled tendon traction, and the reconstruction was completed. A u-nail was also used to increase stability. The AperFix femoral fixation system (Cayenne Med., Inc., Arizona) was used in group 1. In group 2, a fixed-loop femoral fixation device (Artrotek Med., Adana, Turkey) was used.

### 2.2. Postoperative rehabilitation

Patients were mobilized on postoperative day 1. Both groups were allowed to load as much as they could tolerate the crutches, and started to give full load at the end of the 3rd week.^[17]^ No brace was used. Active quadriceps exercises and active knee movements were performed on postoperative day 1. The patients were allowed to return to their normal lives by the 6th postoperative month. Range of motion, clinical scores, and 2-way direct radiographs of the knee were evaluated during follow-up examinations.

**Figure 3.**
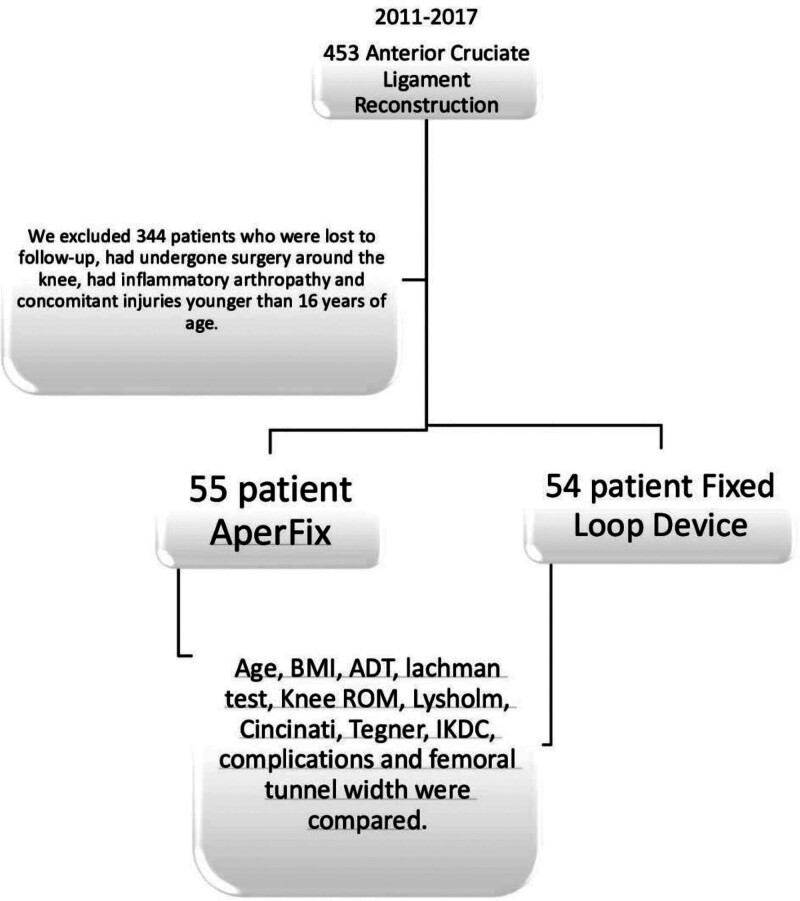
Flow chart of the study. BMI = body mass index, ADT = anterior drawer test, ROM = range of motion, IKDC = International Knee Documentation Committee.

The last outpatient clinic visit was randomly selected by 2 experienced orthopedic teams. Parameters such as age, knee joint flexion-extension degree, anterior drawer test, Lachman test, Lysholm, Cincinnati, Tegner, and International Knee Documentation Committee (IKDC) scores, and incidence of complications were analyzed (Fig. 3).

### 2.3. Radiographic measurements

**Figure 4.**
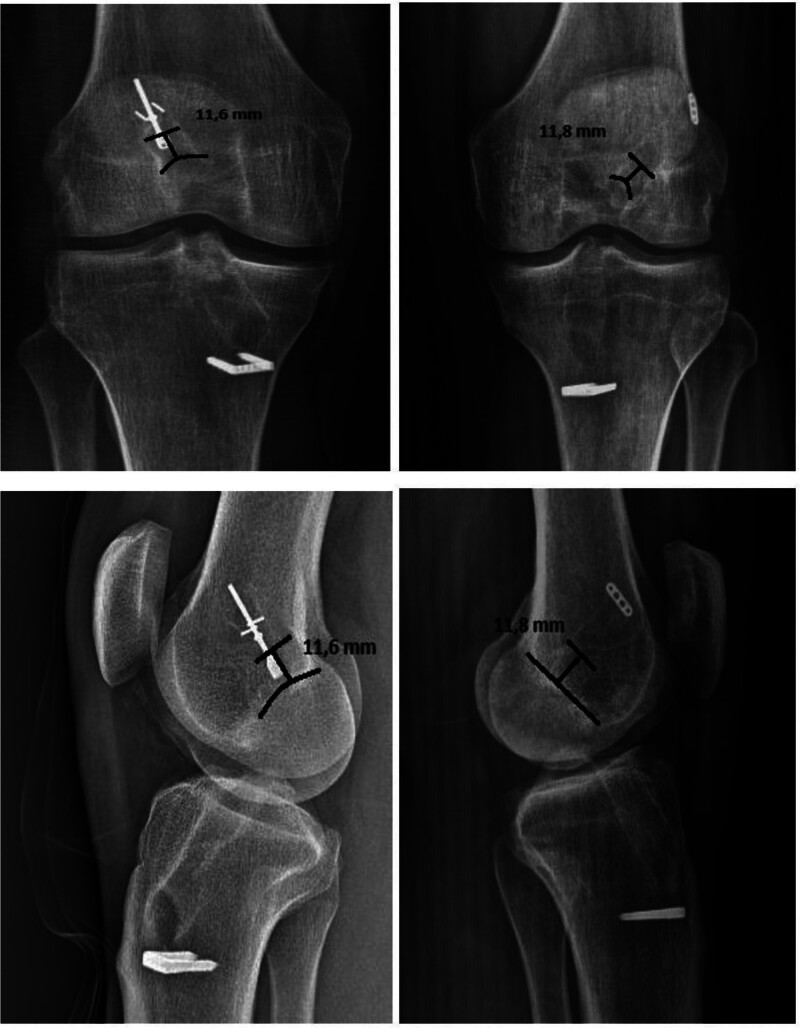
Measurement of femoral tunnel widths.

Femoral tunnel width was measured on anteroposterior and lateral digital radiographs. Because it is a standard digital radiograph, no magnification factor was used because the visual dimension was 1:1. Digital radiographs of patients were evaluated using a computer screen. Radiographic measurements were performed at the last follow-up radiograph. To ensure inter-observer reliability, measurements were performed by 2 different orthopedic surgeons, and the values obtained were averaged. Thus, possible technical errors were minimized. Sclerotic edges near the tunnels were considered as measurement points. The femoral tunnel length was recorded by measuring the distance between the sclerotic area proximal to the tunnel and entry site. In addition, the Fauno and Kaalund method was used to measure the center of the femoral tunnel, and the femoral tunnel widths were recorded^[18]^ (Fig. 4).

### 2.4. Statistical analysis

In this study, sample size was calculated using G*Power statistical programme (ver.3.1.9.7)*. There were 2 groups in the study. Accordingly, in the Independent *T* test experimental design, when the power (power of the test) was 0.80, effect size 0.6 (*t* test effect size value range) and Type-1 error (α) 0.05; ‘a total of 90 samples with a minimum of 45 samples/patient in each group’ was determined. However, in order to ensure the power of the test, the number of samples was increased and 55 samples/patient data in the first group and 54 samples/patient data in the second group were used in our study. The post hoc power value recalculated according to this sample number was found to be 87%. The normal distribution of continuous measurements was confirmed using the Kolmogorov–Smirnov and Skewness–Kurtosis tests. Parametric tests were performed as the data were normally distributed. Variables were described using mean, standard deviation, count (n) and percentage (%). The “Independent *T* test” method was applied for comparing measurements. The “chi-square test (Fisher exact test)” was used to ascertain correlations between the method and categorical variables. The statistical significance level (a) was set at 5%. All analyses were conducted using the SPSS (IBM SPSS for Windows, ver.26) statistical.

## 3. Results

### 3.1. Demographic variables

The results of 109 patients, including 55 in group 1 and 54 in group 2, were evaluated. In terms of average age, sex, body mass index, duration of follow-up, and side distribution, the groups did not show a statistically significant divergence (Table 1).

**Table 1:** Demographic data table.

Femoral fixation method	Number of patients	Gender		Side		Age	BMI
		Male	Female	Right	Left		
AperFix	55	52	3	29	26	27.7	25.35
Endobutton	54	52	2	30	24	27.8	25.83
*P* value		0.925		0.767		0.925	0.412

BMI = body mass index.

### 3.2. Clinical evaluations

No notable disparity was observed between the groups concerning anterior drawer test and positive results from the Lachman test (*P* = .779). There was no statistically significant difference in knee joint flexion-extension degrees between group 1 and group 2 (*P* = .288, *P* = .913). There was also no statistically relevant difference between the Lysholm, Cincinnati, Tegner, and IKDC scores between the groups (*P* = .474, *P* = .969, *P* = .671, *P* = .167) (Table 2). There were no complications in 101 cases and at least 1 complication in 8 cases. These complications include rerupture, infection, and deep vein thrombosis. There was no statistical correlation between complications and fixation method (*P* = .506).

**Table 2:** Comparison of the findings.

	Group 1 (AperFix)		Group 2 (EndoButton)		*P* value
Flexion degree	117.96	(11.64)	120.00	(7.99)	.288
Extension degree	0.83	(4.53)	0.91	(2.37)	.913
Lysholm	83.19	(13.47)	81.35	(13.28	.474
Cincinnati	24.11	(4.60)	24.15	(4.68)	.969
Tegner	5.24	(2.08)	5.42	(2.26)	.671
IKDC	66.69	(11.29)	63.44	(13.00)	.167
Lateral tunnel width	12.34	(0.84)	12.40	(0.91)	.730
Anteroposterior tunnel width	12.31	(0.88)	12.36	(0.84)	.772

Data are presented as mean (SD). *P* value indicates differences among the 2 groups.

IKDC = International Knee Documentation Committee.

### 3.3. Radiographic evaluation

When we analyzed the groups in terms of femoral tunnel width, the mean width at the middle part of the tunnel in the lateral radiographs of the patients in group 1 was 12.34 (±0.84) mm while the mean width at the middle part of the tunnel in the anteroposterior radiographs was 12.31 (±0.88) mm, the mean width at the middle part of the tunnel in the lateral radiographs of the patients in group 2 was 12.4 (±0.91) mm while the mean width at the middle part of the tunnel in the anteroposterior radiographs was 12.36 (±0.84) mm, and the difference in tunnel widths between the methods was not statistically significant (*P* = .730, *P* = .772).

The mean femoral tunnel lengths of the patients were 32.41 mm in group 1 and 31.7 mm in group 2 with no statistically significant difference (*P* = .267).

## 4. Discussion

In this study, we found that the clinical and radiologic results of AperFix and fixed loop femoral fixation methods used in arthroscopic ACL reconstruction were similar. Long-term clinical and radiologic results of the AperFix method, which is missing in the literature, were found to be as good as the other methods.

In research comparing the impact of various graft fixation materials on clinical outcomes following ACL reconstruction, it is still unclear which method is superior.^[7,18–20]^ In a meta-analysis published in 2022 that compared the effectiveness of different graft fixation methods in ACL reconstruction, 1824 patients and 26 different clinical studies were analyzed and it was concluded that the clinical results were similar.^[19]^ However, only 1 study in this meta-analysis included the AperFix method and had short-term follow-up results that were different from those of our study. In another meta-analysis comparing studies using EndoButton, cross-pin, and interference screws for femoral fixation, it was shown that all 3 techniques were not statistically superior.^[20]^ In a meta-analysis conducted by Wang et al^[21]^ in 2020, which included 2 studies in which the AperFix technique was also examined, it was reported that no technique was significantly different from the others in terms of Lysholm score, IKDC category, range of motion, and Tegner score for femoral fixation. However, the follow-up period of the studies related to AperFix in this meta-analysis was <2 years.

Eajazi et al^[22]^ reported a Lysholm score of 96.22 in the AperFix technique and 90.64 in the EndoButton technique in their study with a 2-year follow-up of 96 patients and stated that the score was better in the AperFix technique, but not statistically significant. A possible reason why the Lysholm score reported by the authors was found to be higher than our Lysholm score of 83.19 is the short follow-up period of the patients in their study. The important difference between our study and the present study is that we evaluated the long-term results. In the study by Gormeli et al^[23]^ on the 2-year results of the AperFix technique, Lysholm score was 88.6, Tegner score was 5.3, Cincinati score was 81.3 and it was reported that the AperFix technique gave convincing clinical and radiological results in the early postoperative period. In line with their recommendations, we evaluated the long-term results of the AperFix technique in our study and found similar findings. In a study performed by Sharifzadeh et al^[24]^ in 2017, 1-year results of AperFix and EndoButton fixation methods were compared; no superiority was found between the fixation methods, and it was stated that longer follow-ups were needed. In another study with a mean follow-up period of 40 months, the EndoButton, Transfix, and AperFix methods were compared, and it was reported that the clinical outcomes of arthroscopic ACL reconstruction were not influenced by the use of various femoral fixation methods.^[7]^ Panagopoulos et al^[8]^ indicated that there was no noteworthy distinction in anteroposterior knee stability, tunnel widening or other clinical outcomes between the 2 methods in their prospective study with a 2-year follow-up period. Çiloğlu et al^[25]^ compared the 1-year results of the AperFix and EndoButton techniques in 54 patients and reported similar tunnel enlargement and similar clinical outcomes in both techniques, but they also added that longer follow-up studies are needed.

Digital radiography, magnetic resonance imaging, and computed tomography (CT) have been used to evaluate bone tunnel widening after ACL reconstruction. In the current study, we performed femoral tunnel measurements using digital radiography to avoid excessive radiation exposure and to reduce cost-effectiveness. Marchant et al^[26]^ recommended CT scanning of bone tunnels to evaluate tunnel widening in patients. Fauno and Kaalund^[16]^ indicated a consistent correlation between tunnel widening measurements obtained from plain radiography and those derived from magnetic resonance imaging scans. Webster et al^[27]^ found that digital radiography detected bone tunnel widening at the same rate as CT. We performed femoral tunnel measurements using digital radiography to avoid further radiation exposure and reduce cost-effectiveness. Uzumcugil et al^[16]^ found no difference between the AperFix and EndoButton femoral fixation methods in terms of femoral tunnel widening in controls performed 24 to 36 months after reconstruction. In the current study, we found that femoral tunnel widening was independent of the femoral fixation method.

In many studies in the literature comparing the AperFix and Endobutton (fixed loop device) methods, it was mentioned in the limitations section that the follow-up periods were short and studies with longer follow-up periods should be performed.^[8,22,23,25]^ The most important distinguishing feature of this study is its focus on long-term results with an average follow-up period of 100 months. This makes a valuable contribution to the AperFix technique by filling gaps in the existing literature. The findings address orthopedic surgeons’ concerns regarding the long-term results of the AperFix technique and provide a reliable reference. It also demonstrates that when choosing a femoral fixation method, surgeons can choose techniques that suit their own experience.

## 5. Limitations

Limitations of our study include methodological limitations of the retrospective design, lack of mechanical measurements, lack of preoperative knee function scores, and lack of early postoperative tunnel width measurements. Furthermore, a larger number of patients would have increased the statistical reliability and generalizability of the study. For future research, prospectively designed studies with longer follow-up periods and comparative studies with current fixation techniques are thought to contribute more to the literature.

## 6. Conclusion

AperFix and fixed-loop device fixation methods employed for femoral fixation of hamstring grafts in ACL reconstruction operations have been found to yield similar results when long-term clinical scores, femoral tunnel enlargement, and range of motion were compared. The primary focus of this study is the dearth of long-term research concerning AperFix fixation outcomes in existing literature, highlighting the need for further investigation in this area.

## Author contributions

**Conceptualization:** Sehmuz Kaya, Cihan Adanas, Tulin Turkozu, Ferhat Danisman, Ulan Ismailov.

**Data curation:** Yunus Can Unal, Ferhat Danisman, Ulan Ismailov.

**Formal analysis:** Sezai Ozkan, Ulan Ismailov, Mehmet Ata Gokalp.

**Investigation:** Necip Guven, Ferhat Danisman.

**Methodology:** Sehmuz Kaya, Necip Guven, Cihan Adanas, Ferhat Danisman, Zulkuf Akdemir.

**Project administration:** Sehmuz Kaya, Mehmet Ata Gokalp.

**Resources:** Yunus Can Unal, Tulin Turkozu, Zulkuf Akdemir.

**Software:** Ulan Ismailov.

**Supervision:** Necip Guven, Sezai Ozkan, Cihan Adanas, Tulin Turkozu, Ulan Ismailov, Zulkuf Akdemir.

**Validation:** Sezai Ozkan, Cihan Adanas, Tulin Turkozu, Abdulrahim Dundar, Mehmet Ata Gokalp.

**Visualization:** Yunus Can Unal.

**Writing – original draft:** Sehmuz Kaya, Necip Guven, Abdulrahim Dundar.

**Writing – review & editing:** Sehmuz Kaya, Necip Guven, Abdulrahim Dundar, Mehmet Ata Gokalp.
